# Identifying gene-specific subgroups: an alternative to biclustering

**DOI:** 10.1186/s12859-019-3289-0

**Published:** 2019-12-03

**Authors:** Vincent Branders, Pierre Schaus, Pierre Dupont

**Affiliations:** Université catholique de Louvain - ICTEAM/INGI - Machine Learning Group, Place Sainte Barbe 2, Louvain-la-Neuve, 1348 Belgium

**Keywords:** Gene expression analysis, Biclustering, Gene enrichment analysis, Identification of significant GO terms

## Abstract

**Background:**

Transcriptome analysis aims at gaining insight into cellular processes through discovering gene expression patterns across various experimental conditions. Biclustering is a standard approach to discover *genes subsets* with similar expression across *subgroups of samples* to be identified. The result is a set of biclusters, each forming a specific submatrix of rows (e.g. genes) and columns (e.g. samples). Relevant biclusters can, however, be missed when, due to the presence of a few outliers, they lack the assumed homogeneity of expression values among a few gene/sample combinations. The Max-Sum SubMatrix problem addresses this issue by looking at highly expressed subsets of genes and of samples, without enforcing such homogeneity.

**Results:**

We present here the K-CPGC algorithm to identify *K* relevant submatrices. Our main contribution is to show that this approach outperforms biclustering algorithms to identify several gene subsets representative of specific subgroups of samples. Experiments are conducted on 35 gene expression datasets from human tissues and yeast samples. We report comparative results with those obtained by several biclustering algorithms, including CCA, xMOTIFs, ISA, QUBIC, Plaid and Spectral. Gene enrichment analysis demonstrates the benefits of the proposed approach to identify more statistically significant gene subsets. The most significant Gene Ontology terms identified with K-CPGC are shown consistent with the controlled conditions of each dataset. This analysis supports the biological relevance of the identified gene subsets. An additional contribution is the statistical validation protocol proposed here to assess the relative performances of biclustering algorithms and of the proposed method. It relies on a Friedman test and the Hochberg’s sequential procedure to report critical differences of ranks among all algorithms.

**Conclusions:**

We propose here the K-CPGC method, a computationally efficient algorithm to identify *K* max-sum submatrices in a large gene expression matrix. Comparisons show that it identifies more significantly enriched subsets of genes and specific subgroups of samples which are easily interpretable by biologists. Experiments also show its ability to identify more reliable GO terms. These results illustrate the benefits of the proposed approach in terms of interpretability and of biological enrichment quality. Open implementation of this algorithm is available as an R package.

## Background

Gene expression data is typically represented as a large matrix of gene expression levels across various samples. The study of such data is a valuable tool to improve the understanding of the underlying biological processes. A frequent objective of gene expression analysis is to group genes according to their expression under certain conditions or to group conditions based on the expression of a number of genes. Biclustering, also known as co-clustering, is one of the most common approaches for such a task as it identifies specific subsets of rows and of columns which jointly form homogeneous entries [1, 2].

A substantial number of biclustering methods and applications have been described since the application of biclustering, introduced in [3], to gene expression data analysis [4]. Several biclustering algorithms reviews have been published emphasizing on various characteristics of the biclustering algorithms, applications, or results.

For example, Madeira et al. study in [1] a collection of sixteen biclustering methods and categorize them according to the structures and patterns of biclusters they can find, the methods used to perform the search and the approach used to evaluate the solution. In [5], the survey mentioned above is updated and extended to forty-seven biclustering algorithms. Each method is further categorized based on the use, or not, of evaluation metrics within the search.

Padilha et al. conduct, in [6], a comparative study on seventeen algorithms on a large collection of synthetic and real datasets. They conclude that algorithms only achieved satisfactory results in a specific context, and that best results are obtained by selecting an algorithm depending on the specific task at hand. A similar conclusion is presented in [7], based on results from a comparative study on twelve algorithms on a suite of synthetic datasets and eight real datasets.

A systematic summary of basic and advanced applications of biclustering for biological and biomedical data is presented in [2]. Guidance on the appropriate algorithms and tools to effectively analyze specific data type and to generate valuable biological knowledge is provided.

Biclustering is typically applied on a dataset in the form of a matrix $\mathcal {M}$ where the entry $\mathcal {M}_{i,j}$ represents the value of a specific row *i* (e.g. a gene) obtained for a specific column *j* (e.g. a sample). A bicluster is a submatrix of $\mathcal {M}$ defined by a subset of *selected rows* and a subset of *selected columns*. The selected rows or selected columns need not be contiguous in the original matrix $\mathcal {M}$.

Biclustering algorithms tend to produce biclusters sharing similar expression values, for example by minimizing the variance across the selected genes and selected samples. However, some relevant biclusters may be missed when, due to the presence of a few outliers, they lack the assumed homogeneity of expression values among a few gene/sample combinations.

As an alternative, the max-sum submatrix problem seeks for subsets of rows and of columns with globally high values. In biological terms, one looks for a subset of biomarkers which are, after appropriate normalization, relatively highly expressed among a subset of samples. One could also look for patterns of low expression simply by considering the opposite values of a normalized version of the original matrix. By default, we will look for high expression patterns. Both subsets of selected genes and of selected samples are a priori unknown and must be identified. They form a rectangular, and not necessarily contiguous, submatrix of the original data matrix exactly like biclusters do. Yet, the mathematical criterion used to find such submatrix differs and is less influenced by the presence of some outliers. In the sequel, we use the terms *submatrix* and *bicluster* interchangeably and, depending on the context, they refer to the solution of existing biclustering algorithms or of our own method.

From a biological viewpoint, there might be several biclusters to be identified from the same original data matrix. Indeed, a single gene may participate in multiple pathways which may or may not be co-active under several conditions [1]. Specific genes may also be representative of expression patterns among some samples, while other genes would be more informative for other subsets of samples. In other words, one typically looks at several biclusters which might partially overlap in terms of genes or of samples they contain.

The contributions of this work are:

1. the introduction of the max-sum submatrix optimization problem as an alternative to biclustering,
2. a greedy extension to the CPGC method proposed in [8] to produce several, possibly overlapping, biclusters of maximal sums,
3. a rigorous statistical validation protocol to assess the performances of 6 well-known biclustering methods compared between them and with our proposal,
4. practical experiments on 17 gene expression cDNA microarray datasets from *Saccharomyces cerevisiae* samples under various controlled conditions,
5. a gene enrichment analysis showing that our proposed method outperforms biclustering algorithms to find biologically relevant biclusters,
6. a freely available R package implementing the proposed approach.

### Problem definition

The max-sum submatrix problem consists in finding a rectangular submatrix, not necessarily made of contiguous rows or columns, of a large matrix with maximal sum of the selected entries.

#### The max-Sum submatrix problem (MSSM)

Given a matrix $\mathcal {M} \in \mathbb {R}^{m \times n}$ consisting of *m* rows and *n* columns, let $\mathcal {R} = \lbrace 1,\dots, m \rbrace $ and $\mathcal {C} = \lbrace 1,\dots, n \rbrace $ be index sets for rows and for columns respectively, find the max-sum submatrix (*I*^∗^;*J*^∗^), with ${I}^{*} \subseteq \mathcal {R}$ and ${J}^{*} \subseteq \mathcal {C}$, such that:

1 $$\begin{array}{*{20}l} (I^{*}; J^{*}) = {\underset{I \subseteq \mathcal{R}, J \subseteq \mathcal{C}}{\arg\max}}\; f(I,J) = \underset{I \subseteq \mathcal{R}, J \subseteq \mathcal{C}}{{\arg\max}} \sum_{i \in {I}, j \in {J}} \mathcal{M}_{i, j}. \end{array} $$

#### Interpretation

The data matrix typically represents gene expression values in a continuous range, for instance on a logarithmic scale and properly normalized: negative values, respectively positive values, represent expression values below, respectively above, a threshold *θ*. For example, *θ* may correspond to the median expression level over the whole data matrix, or a row-specific value representing the average expression level of a gene across all samples. After such normalization, positive values are considered as the interesting ones. By default, they correspond to the high levels of expression one is interested to find in the data matrix. One could also look for low levels of expression by replacing such a normalized matrix $\mathcal {M}$ by its opposite $-\mathcal {M}$. In any case, the *objective function* *f*(*I*,*J*) is the sum of the entries of a submatrix (*I*;*J*). The maximization of this objective rewards, respectively penalizes, matrix entries with positive, respectively negative, values.

**Fig. 1:**
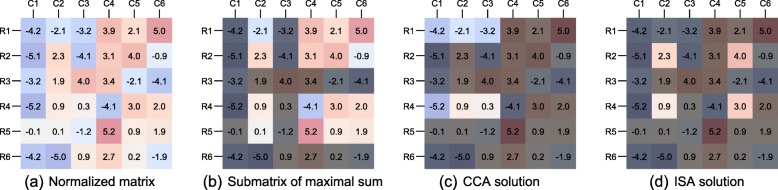
Toy example. **a** Illustration of a full, normalized, matrix, **b** the associated submatrix of maximal sum, **c** bicluster returned by CCA and **d** bicluster return by ISA

Figure 1a depicts a toy example of such a normalized data matrix. Positive values (in red) are considered to have high expression levels and negative values (in blue) correspond to low expression levels. Figure 1b represents the optimal solution to the maximal sum objective. It defines a specific rectangular submatrix, or bicluster, of genes and samples, maximizing the sum of its entries. It can include a few outliers in terms of high expression levels. For example, the -4.1 entry (row 4, column 4) is included in the optimal solution because such a low value is compensated along its row and its column by other positive values, hence all selected rows and selected columns contribute positively to the objective function. In contrast, as one looks for a *rectangular* submatrix, a positive entry may be excluded from the optimal solution if it is penalized by the presence of negative values along its row and its column. This is the case, for example, for the entry 4.0 in row 3 and column 3 of this toy example.

Figure 1c and d represents the results obtained with two different biclustering algorithms, namely CCA and ISA (further described in the “Methods” section), starting from the same toy example (Fig. 1a). Both their solutions strongly differ from the one represented in Fig. 1b. In particular, the CCA solution includes many negative entries as they imply a lower variance along selected rows and selected columns. In contrast, the ISA solution only includes positive entries but is missing several genes and samples that should arguably be selected as in Fig. 1b. Our experimental results reported in the “Results” section illustrate the benefits of the proposed approach to extract biologically relevant gene subsets.

Finding a submatrix of maximal sum, as formalized in problem (1), is an NP-hard combinatorial optimization problem. This can be shown from a reduction to the maximum edge weight biclique problem [9], by considering the rows and columns of the original matrix as the two sets of nodes of a bipartite graph. This problem is difficult to solve, especially in gene expression analysis, as one is typically interested in solving it for large instances made of thousands of genes (rows) and possibly hundreds of samples (columns). Polynomial algorithms have been proposed in the restricted case of finding a submatrix made of contiguous rows and contiguous columns of the original matrix [10–12]. Such a restriction is however not justified in the general context of transcriptomics since it would require to know in advance unique and specific orders in which the genes and the samples can be clustered.

Our previous work [8] presents several algorithms to address problem (1) without any restriction. They include a mixed integer linear programming (MILP) and two constraint programming (CP) approaches. The first CP method combined with large neighborhood search is an improved version of the work proposed in [13]. Gene expression analysis results were already reported in this earlier work but the proposed approach looks for fully disjoint biclusters. We argue that some overlap may exist between various expression patterns extracted from the same data matrix. The second method, denoted CPGC, includes a global constraint and proved, in our previous work, to be the most efficient one to solve problem (1). We study here its applicability to discover several, and possibly overlapping, biclusters from gene expression data and we show its benefits compared to existing biclustering algorithms.

## Results

Table 1 gives a global overview of the ability of various algorithms to find significantly enriched biclusters among 35 gene expression datasets from human tissues and *Saccharomyces cerevisae*. The eight algorithms and 35 gene expression datasets considered in this work are detailed in the “Methods” section. The K-CPGC algorithm clearly outperforms the other approaches in this global overview: it is the best in terms of the number of enriched biclusters found. Some algorithms are only able to produce a limited number of distinct biclusters, even less enriched ones. This is due to the specifics of each algorithm. For instance, several random initializations used by ISA do not guarantee to find distinct solutions. Plaid only returns biclusters that offers a better fit to their underlying statistical model than those obtained through random permutations of the original matrix. As for K-CPGC, a slight increase of the threshold *θ* would lead to producing more biclusters while constraining further the objective of finding high expression patterns. The reported setting looks sound anyway as the prescribed number of 10 biclusters for each dataset is very close to being found with this approach.

**Table 1:** Total number of identified and enriched biclusters

Algorithm	Biclusters	Enriched biclusters
CCA	349	108
ISA	163	90
K-CPGC	342	**177**
Plaid	102	57
QUBIC	269	107
Spectral	147	44
xMOTIFs	309	60
CPGC	35	35

Results reported for each algorithm on the 35 gene expression datasets from human tissues and *Saccharomyces cerevisae*. The defined target is *K*=10 biclusters for each dataset, for a maximum of 350 biclusters overall. A bicluster is considered significantly enriched if the subset of genes it contains is associated to at least one GO term with an FDR corrected *p*-value below 5%

A non-parametric Friedman test [14] is routinely used in the machine learning literature to assess the relative performance of various classification algorithms across several datasets [15]. We adopt here the same methodology to compare biclustering algorithms and our own K-CPGC method. For each dataset, the algorithms under study are ranked according to the number of enriched biclusters they return. Table 2 reports the number of enriched biclusters identified per dataset by each algorithm and its associated rank. The last row reports the average rank *R*_*A*_ of algorithm *A* over all datasets. The Friedman statistic has a $\chi ^{2}_{F}$ distribution with *ν*−1 degrees of freedom where *N* is the number of datasets and *ν* the number of algorithms being tested:

2 $$\begin{array}{*{20}l} \chi^{2}_{F} = \frac{12N}{\nu(\nu+1)} \left[ \sum_{A=1}^{\nu} R^{2}_{A} - \frac{\nu(\nu+1)^{2}}{4} \right] \end{array} $$

**Table 2:** Number of enriched biclusters found by each algorithm on each dataset

dataset	CCA	ISA	K-CPGC	Plaid	QUBIC	Spectral	xMOTIFs	CPGC
1	1 (5.0)	**7** (1.0)	6 (2.0)	1 (5.0)	0 (7.5)	0 (7.5)	2 (3.0)	1 (5.0)
2	0 (7.5)	**8** (1.0)	6 (2.0)	1 (5.0)	1 (5.0)	0 (7.5)	2 (3.0)	1 (5.0)
3	1 (6.0)	**8** (1.0)	7 (2.0)	1 (6.0)	5 (3.0)	0 (8.0)	3 (4.0)	1 (6.0)
4	1 (5.5)	2 (2.5)	1 (5.5)	2 (2.5)	0 (8.0)	**5** (1.0)	1 (5.5)	1 (5.5)
5	2 (4.0)	**6** (1.5)	2 (4.0)	1 (6.5)	**6** (1.5)	0 (8.0)	2 (4.0)	1 (6.5)
6	1 (5.5)	**7** (1.0)	5 (2.0)	2 (4.0)	0 (7.5)	0 (7.5)	3 (3.0)	1 (5.5)
7	2 (5.0)	3 (4.0)	**8** (1.0)	1 (7.0)	7 (2.5)	7 (2.5)	1 (7.0)	1 (7.0)
8	3 (5.0)	**8** (1.5)	**8** (1.5)	1 (7.5)	6 (3.0)	5 (4.0)	2 (6.0)	1 (7.5)
9	1 (7.0)	2 (4.5)	**7** (1.0)	1 (7.0)	6 (2.5)	6 (2.5)	2 (4.5)	1 (7.0)
10	1 (5.0)	1 (5.0)	**5** (1.0)	1 (5.0)	0 (8.0)	2 (2.0)	1 (5.0)	1 (5.0)
11	0 (7.5)	1 (4.5)	**4** (1.5)	0 (7.5)	**4** (1.5)	1 (4.5)	1 (4.5)	1 (4.5)
12	2 (3.5)	**8** (1.0)	7 (2.0)	0 (7.5)	2 (3.5)	0 (7.5)	1 (5.5)	1 (5.5)
13	0 (7.0)	**3** (1.5)	**3** (1.5)	0 (7.0)	2 (3.5)	0 (7.0)	2 (3.5)	1 (5.0)
14	2 (4.5)	3 (2.5)	1 (6.5)	0 (8.0)	2 (4.5)	**10** (1.0)	3 (2.5)	1 (6.5)
15	**3** (2.0)	**3** (2.0)	2 (4.5)	0 (8.0)	**3** (2.0)	2 (4.5)	1 (6.5)	1 (6.5)
16	1 (5.5)	**8** (1.5)	**8** (1.5)	0 (7.5)	4 (3.0)	0 (7.5)	2 (4.0)	1 (5.5)
17	0 (7.0)	**3** (2.0)	1 (4.5)	0 (7.0)	**3** (2.0)	0 (7.0)	**3** (2.0)	1 (4.5)
18	2 (2.0)	1 (4.0)	**3** (1.0)	0 (7.0)	1 (4.0)	0 (7.0)	0 (7.0)	1 (4.0)
19	8 (2.5)	0 (7.0)	**9** (1.0)	6 (4.0)	8 (2.5)	0 (7.0)	0 (7.0)	1 (5.0)
20	6 (2.0)	3 (4.5)	**10** (1.0)	3 (4.5)	4 (3.0)	0 (8.0)	2 (6.0)	1 (7.0)
21	2 (4.0)	1 (6.5)	**8** (1.0)	4 (2.0)	2 (4.0)	0 (8.0)	2 (4.0)	1 (6.5)
22	6 (2.0)	1 (6.5)	**8** (1.0)	0 (8.0)	3 (4.5)	5 (3.0)	3 (4.5)	1 (6.5)
23	2 (2.5)	0 (7.0)	**4** (1.0)	0 (7.0)	2 (2.5)	0 (7.0)	1 (4.5)	1 (4.5)
24	**4** (1.5)	0 (6.5)	**4** (1.5)	0 (6.5)	0 (6.5)	0 (6.5)	1 (3.5)	1 (3.5)
25	**5** (1.5)	0 (7.0)	**5** (1.5)	3 (3.0)	0 (7.0)	0 (7.0)	1 (4.5)	1 (4.5)
26	**4** (1.5)	0 (7.5)	**4** (1.5)	2 (4.5)	2 (4.5)	0 (7.5)	3 (3.0)	1 (6.0)
27	**4** (1.0)	1 (6.5)	3 (2.0)	2 (4.0)	2 (4.0)	0 (8.0)	2 (4.0)	1 (6.5)
28	**5** (1.0)	0 (7.0)	4 (2.0)	2 (3.5)	2 (3.5)	0 (7.0)	0 (7.0)	1 (5.0)
29	3 (3.5)	1 (6.0)	**6** (1.0)	3 (3.5)	4 (2.0)	0 (8.0)	1 (6.0)	1 (6.0)
30	**5** (1.0)	0 (7.5)	2 (2.5)	1 (5.0)	1 (5.0)	0 (7.5)	2 (2.5)	1 (5.0)
31	4 (2.5)	1 (5.5)	**5** (1.0)	4 (2.5)	1 (5.5)	0 (8.0)	1 (5.5)	1 (5.5)
32	**8** (1.5)	0 (7.5)	5 (4.5)	7 (3.0)	**8** (1.5)	0 (7.5)	5 (4.5)	1 (6.0)
33	6 (3.0)	0 (8.0)	7 (2.0)	3 (4.0)	**9** (1.0)	1 (6.0)	1 (6.0)	1 (6.0)
34	6 (2.0)	0 (7.5)	4 (3.0)	3 (4.5)	**7** (1.0)	0 (7.5)	3 (4.5)	1 (6.0)
35	**7** (1.0)	0 (6.5)	5 (2.0)	2 (3.0)	0 (6.5)	0 (6.5)	0 (6.5)	1 (4.0)
avg. rank	3.7	4.5	**2.1**	5.4	3.9	6.2	4.7	5.6

Numbers in parentheses are the associated ranks. In case of ties, average ranks are assigned. The last row corresponds to the algorithm ranks averaged over the 35 datasets. Best performances are highlighted in bold. It is observed that all enriched biclusters have different GO enrichment. Note that CPGC is the original algorithm identifying a single submatrix of maximal sum per dataset

The results presented in Table 2 lead to reject the null hypothesis of no difference between the *ν*=8 algorithms over *N*=35 datasets with an associated *p*-value equal to 1.33×10^−11^. It should be highlighted that some algorithms present performances discrepancies regarding the data collections. Namely, ISA, QUBIC and Spectral present higher enrichment performances on the Human tissues collection than on the Yeast collection. Each of the other approaches provides comparable performances on both collections of datasets.

**Fig. 2:**
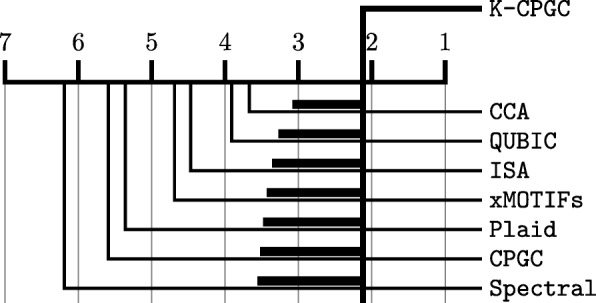
Critical difference of ranks. Comparison between the average rank of each algorithm over *N*=35 datasets, with a 5% level of significance and Hochberg’s correction for multiple testing

We proceed with a post hoc test, the Hochberg’s sequential procedure [16], to determine whether K-CPGC significantly outperforms the other algorithms. Figure 2 reports a diagram of critical differences between the ranks of the various algorithms. The horizontal lines in **bold** represent the differences between ranks that are required for statistical significance. Such intervals increase as more approaches are included in the comparison following the Hochberg’s correction for multiple testing. In conclusion, K-CPGC has a significantly better rank compared to all other approaches.

**Fig. 3:**
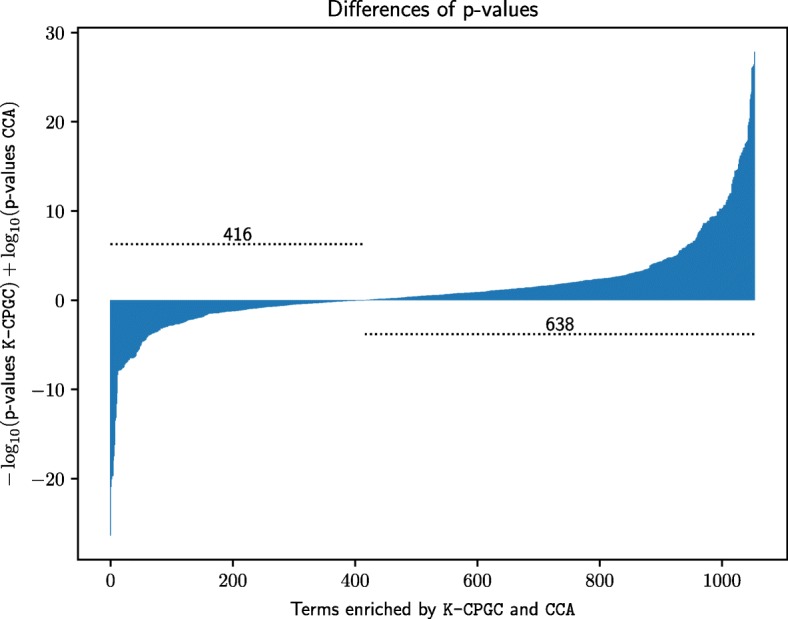
Comparison of K-CPGC and CCA*p*-values for enriched GO terms. This figure presents the (logarithmic) ratio of corrected *p*-values associated to each GO term identified by both K-CPGC and CCA on all the 35 datasets. Positive values (638 GO terms) are in favor of K-CPGC

The analysis so far has been focusing on the number of biclusters for which the subset of genes they contain is associated with *at least one significant GO term*. Since K-CPGC and CCA are the best methods according to this analysis, one looks now at *all significant GO terms* identified by both algorithms on all 35 datasets. Figure 3 reports the difference metric between *p*-values of these GO terms according to equation (4). It shows that K-CPGC outperforms CCA in this regard since it exhibits a positive difference in 638 out of 1054 cases. In other words, K-CPGC identifies gene subsets which are generally estimated more significant as they correspond more often to lower *p*-values.

**Fig. 4:**
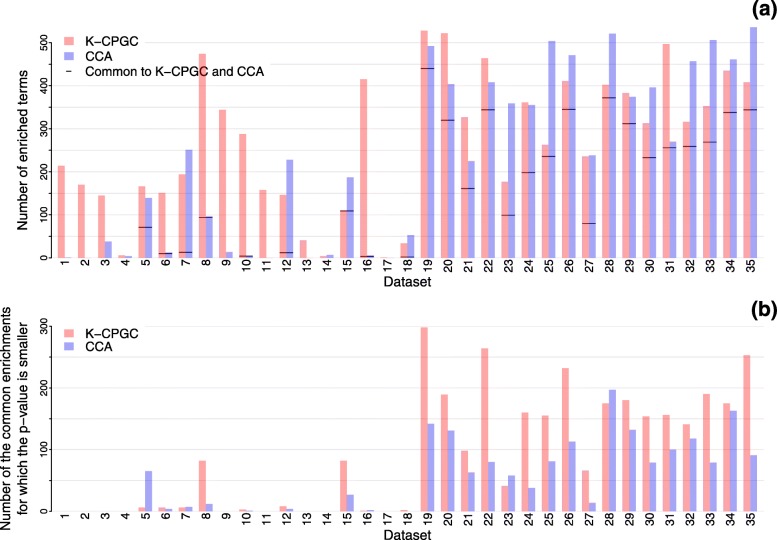
Comparison of terms enriched by K-CPGC and CCA for each of the 35 datasets. **a** Number of terms enriched by K-CPGC and CCA for each of the 35 datasets. The horizontal line reports the number of terms that are enriched by both approaches. **b** Number of times that the adjusted *p*-value of a term found by an algorithm is smaller than the adjusted *p*-value of the term found by the other algorithm. Only terms enriched by both algorithms are considered

To complement the analysis, we evaluate the *p*-values differences on a per dataset basis. Comparing *p*-values of terms found by K-CPGC and CCA would have little sense whenever the enriched terms differ completely between both approaches, however. As a consequence, we first report in Fig. 4a the number of terms found by the two approaches, as well as the number of terms that are common to both approaches. Figure 4b reports the number of the terms common to K-CPGC and CCA for which an algorithm presents a smaller *p*-value as compared to the other algorithm. It shows that when K-CPGC and CCA return several GO terms in common, the significance of such terms is typically better with our K-CPGC approach (see Fig. 4b). When no such overlap exists between the enriched terms reported by both approaches, this is essentially due to the fact that CCA hardly returns any enriched terms (see Fig. 4a). Such a per dataset analysis further supports the benefits of our proposed method.

We further analyze the actual gene subsets identified by K-CPGC from *Saccharomyces cerevisae* samples to check whether the 20 most significantly enriched GO terms it identifies in each dataset are consistent with the controlled conditions under which these experiments were conducted. The full analysis is detailed in the supplementary materials associated with this manuscript [see Additional file 1]. It shows that the GO terms identified in the first four datasets, representative of cell cycles, are associated with some form of biogenesis, including ribosome, RNA, peptide and macromolecules synthesis. The GO terms identified in the next 12 datasets are indeed associated with various forms of response to stress-induced environments, including many representatives of the response to the stimulus, oxidation-reduction processes, and cellular responses to stress. Some GO terms also refer to generic responses to stress which are less specific to the controlled condition. For example, in the *complete DTT* dataset, many GO terms relate to alteration in the general patterns of protein biosyntheses as reported by Miller et al. [17]. The last experiment related to yeast sporulation includes GO terms referring to cell cycle, sporulation, and reproductive processes.

While this work focuses on the biological relevance of identified submatrices, it must be stressed that K-CPGC usually finds the best solutions in less than a minute^1^. On average, K-CPGC requires 14.7 s, the median being equal to 1.7 s. The longest run is performed within 195.7 s on dataset 18 (Yeoh-v1).

## Discussion

The experiments and results reported in this work show that the K-CPGC method outperforms six well-known biclustering algorithms to identify biologically relevant gene subsets among subgroups of samples. The various algorithms are compared essentially based on their ability to return gene subsets which are associated with significantly enriched GO terms. One could consider that such a performance assessment validates only part of the results as it focuses on the genes (rows) and does not validate *a posteriori* the identified subgroups of samples (columns). This is actually a common limitation of the assessment of biclustering methods from gene expression data [6]. For the *Saccharomyces cerevisae* experiments reported here, there is no gold standard in terms of subgroups of samples to be identified. Yet, these subgroups of samples are, at least indirectly, validated because their components directly influence the subsets of genes which are returned. This is particularly clear for the CPGC approach as one looks for a rectangular submatrix of maximal sum and the returned genes are directly constrained by the selected samples in such a submatrix. This is also true for biclustering algorithms since, for instance, they look for homogeneous expression patterns both across rows and columns. Notwithstanding, in a medical context, for example, the actual samples are typically associated with specific patients. In such a case, direct validation of the identified subgroups of samples could be performed by comparing these subgroups with actual clinical annotations. Interpreting the evaluation of unsupervised method on their ability to recover an expected structure is difficult, however. As an illustrative example, Padilha et al. [6] evaluated the ability of several biclustering algorithms to recover the predefined sample classes. They showed that the best methods are biased towards methods that force every row and every column to be biclustered.

The proposed K-CPGC method could be considered as including two control parameters: the number *K* of biclusters one looks for and the threshold *θ* defining the level of expression above which interesting patterns are searched. In the present work, we fix *θ* to the 75th percentile of expression values and we argue that this is a reasonable choice to find high expression patterns. Yet, the user may be interested to play with this parameter as it influences indirectly the sizes of the biclusters found. In the limit, if *θ* is set below the minimal expression value, all entries of the normalized matrix will be positive and the solution to the maximal sum problem is trivially identified as the full matrix. Similarly, if *θ* is set above the maximal expression value, all entries of the normalized matrix become negative and the optimal solution is the empty matrix. An intermediate *θ* value between these extreme cases is typically chosen. For a fixed data matrix *increasing* *θ* tends to produce *smaller* biclusters. The actual bicluster sizes found is difficult to predict exactly, as it also depends on the actual distribution of expression value in the matrix, but the analyst may easily play with *θ* to find biclusters of interest.

In the present study, for a fair comparison between all algorithms, we fix the maximal number of biclusters to be found to *K*=10. In practice, however, this is not a critical choice since the analyst can start with *K*=1 and use the proposed gene enrichment analysis to check whether the successive biclusters returned by increasing *K* are still significantly enriched [see Additional file 2]. This could even be automated in a script and the actual value of *K* automatically determined in this way.

As already mentioned in the “Interpretation” section, the K-CPGC approach can also be used to find low expression patterns instead of high-level ones simply by considering the opposite of the normalized data matrix. These are two obvious possibilities but it is straightforward to generalize this approach. For instance, if one would be interested in finding patterns of average expression values (neither over-expressed nor under-expressed), one can easily transform the original matrix to a new one, e.g. according to a Gaussian or RBF kernel, in which a higher value would represent an original entry closer to the average expression value. This average (or median) value can be computed overall, row-wise or column-wise. Countless variants are easy to define and illustrate the flexibility of this approach.

## Conclusions

We propose a novel algorithm, K-CPGC, to find *K* non-redundant and possibly overlapping submatrices of maximal sum from a large gene expression matrix. The returned solutions have the same bi-dimensional structure as biclusters produced by existing biclustering algorithms. Yet, the mathematical objective is different and more explicitly optimized with the proposed methodology. Indeed, the role of a matrix entry $\mathcal {M}_{i,j}$ in a submatrix is clear: its contribution to the decision of including gene *i* and sample *j* in the submatrix is $\mathcal {M}_{i,j}$. It follows that the contribution of each gene in the definition of a gene subset, respectively each sample, can be easily computed as the sum of matrix entries for each of the selected samples, respectively genes.

Through enrichment analysis performed on 35 gene expression datasets from human tissues and *Saccharomyces cerevisae* samples, we show that K-CPGC outperforms biclustering algorithms when looking for biologically relevant gene subsets. Not only is our approach efficient, but it also identifies more enriched biclusters than other biclustering methods. The K-CPGC approach provides stronger results (lower *p*-values of gene subsets or GO terms) than these alternative algorithms. These results illustrate the benefits of the proposed approach in terms of biological enrichments and biological relevance.

The K-CPGC is, however, not limited to gene expression analysis. For example, Liu and Wang [18] use a drug activity dataset consisting of a matrix of 10,000 compounds with 30 features for each compound. The K-CPGC algorithm could be used to identify subsets of compounds presenting highly valued entries in subsets of features.

This method has the potential to find relevant gene subsets across various -omics technologies since, unlike biclustering algorithms, it does not look for homogeneous gene expression values. The specific search order it follows could also be easily adapted to discover small relevant submatrices rather than large biclusters, hence focusing on rare but relevant expression patterns.

The K-CPGC method and the biclustering algorithms it is compared to are *unsupervised* methods since they do not require any particular annotation of the analyzed samples. A different and interesting setting arises when the samples, or at least a fraction of them, are labeled according to various conditions or clinical variables. In such a context, a new objective would be to identify subsets of genes that are maximally relevant to discriminate between subsets of samples from different conditions.

## Methods

This section briefly presents six biclustering algorithms frequently cited in the literature and for which software implementations are publicly available [6, 7, 19–22]. Next, we present our own constraint programming approach, CPGC, to identify a submatrix of maximal sum and its extension to extract *K* submatrices. Our evaluation protocol, including the data collection and experimental setup, is also detailed.

### Biclustering algorithms

*Cheng and Church’s Algorithm* (CCA) is based on iteratively adding or removing rows and columns to a current bicluster in order to minimize the variance within it [4]. The variance in a bicluster (*I*;*J*) is evaluated as a mean squared residue $\text {MSR} = \frac {1}{\vert I \vert \vert J \vert } \sum _{i \in I, j \in J} (\mathcal {M}_{ij} - \mathcal {M}_{iJ} - \mathcal {M}_{Ij} + \mathcal {M}_{IJ})^{2}$, where $\mathcal {M}_{i,j}$ is the average of the *i*th row in the bicluster, $\mathcal {M}_{i,j}$ the average of the *j*th column, and $\mathcal {M}_{i,j}$ the average of all elements in the bicluster. A parameter *δ* defines a threshold of maximum MSR for a bicluster to be accepted. The identification of multiple biclusters is achieved iteratively by replacing all entries of the previously identified bicluster(s) by random values within the range of the original data matrix.

*Conserved Gene Expression Motifs* (xMOTIFs) finds biclusters with simultaneously conserved genes in subsets of samples in a discretized data matrix [23]. Each discretized entry corresponds to a continuous range of expression values from the original matrix. Genes are considered conserved across a subset of samples if the discretized expression values are identical. This approach greedily searches for a largest xMOTIF starting from various random seeds. When such an xMOTIF is found, the corresponding samples are removed from the original matrix and the whole process is iterated. This approach is thus constrained to return biclusters without overlap between the respective samples they contain.

*Iterative Signature Algorithm* (ISA) starts from a randomly selected bicluster and greedily adds or removes columns and rows till reaching some prescribed minimal average value *T*_*C*_ (*T*_*R*_) across the selected columns (rows) [24]. Several biclusters can be found by restarting from another randomly selected bicluster.

*QUalitative BIClustering* (QUBIC) discretizes the original matrix and builds a graph where each node corresponds to a gene, and each edge weight is the number of samples for which two genes have the same nonzero discretized value. It then searches for biclusters corresponding to heavy subgraphs [19].

*Plaid* fits a generative statistical model with *K* components from which each entry $\mathcal {M}_{ij}$ of the original matrix is assumed to have been generated [25].$ \mathcal {M}_{ij} = B + \sum _{k=1}^{K} (\mu _{k} + \alpha _{ik} + \beta _{jk})\rho _{ik}\kappa _{jk} + \varepsilon _{ij} $ where *B* is a background level, *μ*_*k*_ is a specific bicluster effect, *α*_*ik*_ and *β*_*jk*_ are row and column effects, *ρ* and *κ* are cluster memberships respectively along the rows and the columns, *ε* is a random noise. The Plaid algorithm fits such an additive model by minimizing a mean square error between the modeled and observed data [26]. This algorithm may actually return less than *K* biclusters because a specific bicluster is returned only if it offers a better fit (= a lower residue) than biclusters found from random permutations of the original matrix.

*Spectral* relies on singular value decomposition to cluster genes and samples simultaneously after a specific normalization of rows and columns [27]. It looks for distinctive checkerboard patterns which form biclusters including contiguous rows and contiguous columns. The net result is a set of biclusters of low variance such that each gene and each sample exactly belong to a single bicluster.

### The CPGC method

**Fig. 5:**
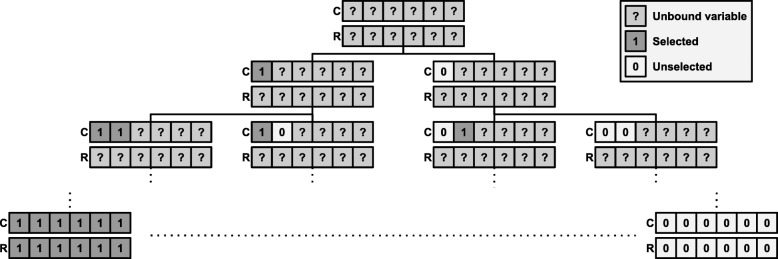
Search tree. This figure illustrates the search tree defined on the set of possible submatrices. A question mark refers to an unbound variable that can be equal to 0 or 1

The CPGC method has been introduced in [8] to solve the Max-Sum Submatrix Problem (1) through constraint programming (CP). A solution to the MSSM problem is represented by two vectors of boolean decision variables *R*=(*R*_1_,…,*R*_*m*_) for the rows and *C*=(*C*_1_,…,*C*_*n*_) for the columns, with *R*_*i*_∈{0,1} and *C*_*j*_∈{0,1}. When a decision variable is equal to 1, its corresponding row or column is selected in the solution. When it is equal to 0, its corresponding row or column is not part of the selected submatrix. The algorithm searches through the space of possible variable assignments in the form of a tree as depicted in Fig. 5. Initially, at the root, all decisions variables are unbound and the algorithm explores such a tree in a depth-first fashion. Any configuration with no unbound variable defines a specific submatrix and is called a feasible solution. The goal is to find an optimal solution, *i.e.* a solution of maximal sum, among the feasible solutions.

#### Implicit search space

Any feasible solution to the problem is defined by a complete assignment of the selected rows *and* columns. Such a complete assignment defines a specific rectangular submatrix ($I\subseteq \mathcal {R}; J \subseteq \mathcal {C}$) of the original matrix. The full search space includes $\left (2^{\vert \mathcal {R}\vert }-1\right) \times \left (2^{\vert \mathcal {C}\vert } - 1\right)$ non-empty feasible solutions. Yet, for any full assignment of the columns (no unbound *C*_*j*_ variable), the optimal assignment of the rows can be directly computed. Indeed, for any of the two dimensions being fixed, optimization along the other dimension is straightforward since it amounts to select only the subset of entries whose contribution is positive. For a fixed subset of columns $J \subseteq \mathcal {C}$, the optimal subset of rows $I^{*}_{J} \subseteq \mathcal {R}$ that maximizes the objective value is identified as $I^{*}_{J} = \lbrace i \in \mathcal {R} \; \vert \; \sum _{j \in J} \mathcal {M}_{i,j} \geq 0 \rbrace $.

In the gene expression analysis context, with order(s) of magnitude more rows (genes) than columns (samples), the actual search space is explored only over the column assignments. For each of the $O(2^{\vert \mathcal {C} \vert })$ column assignments, the optimal subset of rows can then be computed in linear time.

#### Efficient search and filtering

The CPGC algorithm includes several refinements to speed up the search for an optimal solution. It uses a branch and bound strategy to avoid the exploration of proven suboptimal solutions. A CP algorithm usually updates a best so far lower bound to the objective whenever it reaches a leaf of its search tree, that is when every decision variable is bound. For the MSSM problem, any partial assignment of the decision variables can be extended as a complete solution for which the unbound variables are set to 0. It is thus possible to update the best lower bound so far at each node of the search tree, which can improve the filtering of suboptimal solutions.

The complexity of this approach is defined by the number of nodes explored and the complexity of the methods executed at each node. The CPGC approach explores $O\left (2^{\vert \mathcal {C} \vert }\right)$ nodes, or possible assignments of column variables. The time complexity of the methods performed at each node of the search tree is in $O(\vert \mathcal {R} \vert \times \vert \mathcal {C} \vert)$. The global time complexity of CPGC is therefore in $O\left (2^{\vert \mathcal {C} \vert } \times {\vert \mathcal {R} \vert } \times \vert \mathcal {C} \vert \right)$.

The space complexity of the nodes is in $O(\vert \mathcal {R} \vert + \vert \mathcal {C} \vert)$. The number of nodes to maintain effectively is in $O(\vert \mathcal {C} \vert)$, by virtue of the depth-first search exploration strategy. The global space complexity of CPGC is therefore in $O\left (\vert \mathcal {C} \vert \times \left (\vert \mathcal {R} \vert + \vert \mathcal {C} \vert \right) \right)$.

These bounds on the space and time complexities do not take into account the substantial reduction of the search space induced by the filtering procedures. In experiments with instance matrices of 10,000 rows by 1000 columns, the best solutions are found within short periods of time, usually less than 10 s^1^. Moreover, providing more time (up to 1000 s) never improves the objective value.

These results suggest that CPGC is scalable to tackle reasonably large problems from biological to biomedical domains. The interested reader is invited to consult [8] for further technical details about this approach.

### Identification of *K* biclusters

The CPGC algorithm looks for a single submatrix of maximal sum from an original data matrix while there might be several biclusters to be identified. In gene expression analysis, the same gene may indeed participate in multiple pathways. Hence one would like to identify *K* biclusters with possible overlaps between them. The control parameter *K* must be chosen by the data analyst (e.g. *K*=10) but, as illustrated in the “Results” section, a biological interpretation of the biclusters found may help in this regard. Formally, any row and any column of the original data matrix may belong to zero, one up to *K* biclusters. Hence, each decision variable can now take 2^*K*^ values. The extension of the MSSM problem to identify *K* solutions would thus lead to a search space containing $O\left (2^{K^{\vert \mathcal {R} \vert }} \times 2^{K^{\vert \mathcal {C} \vert }}\right)$ feasible solutions. A completed assignment of the column variables does not help as in the “Implicit search space” section. Indeed, each decision for row *i* in submatrix *k* depends on the decisions on row *i* for the *K*−1 other submatrices. Consequently, one can no longer hope to find optimal solutions in a reasonable time from real gene expression datasets. Instead, we propose to follow a greedy strategy as commonly adopted in several biclustering algorithms [1, 4, 5].

A first submatrix is found by solving the optimization problem (1) with CPGC. Next, the values of the selected entries in this solution are replaced in the original matrix by zeros. A zero value is indeed neutral with respect to the maximal sum objective. In other words, any particular entry that has already been selected can again be selected but without any benefit nor loss in the objective value. Such a strategy allows for a possible overlap between several biclusters, neither forcing such overlap nor discarding it a priori. This process can be iterated till producing *K* biclusters.

The time complexity of the method is computed as *K* times the complexity of the CPGC subroutine. The greedy procedure does not alter the space complexity. Identifying *K* submatrices with a large total sum is performed within a reasonable time (in the order of a minute), which is unsurprising given the performances of the CPGC subroutine.

An implementation of this greedy algorithm, called K-CPGC, is freely available as an R package from https://github.com/vbranders/mssm.

### An evaluation study on human tissues and on *Saccharomyces cerevisiae*

In this study, we look for biologically relevant biclusters computed from thirty-five publicly available gene expression microarray datasets. The first 18 datasets were obtained from human tissues using single-channel Affymetrix chips (Affy), proposed and preprocessed by de Souto et al. [28]. Similarly to the latter work, expression values are transformed prior to further analyzes: $\mathcal {M}^{*}_{i,j} \leftarrow \log _{2}(\mathcal {M}_{i,j}/m_{i})$ where *m*_*i*_ is the median of row *i* and $\mathcal {M}^{*}_{i,j}$ is the value in row *i* and column *j* after transformation. The subsequent 17 datasets, proposed and preprocessed by [29], were obtained from *Saccharomyces cerevisiae* samples under various controlled conditions using double-channel cDNA (cDNA) technology. These expression values are left unaltered. Table 3 summarizes this collection by reporting the number of genes and samples measurements in each dataset.

**Table 3:** Data collection summary

	Name	Chip	Genes	Samples	Organism	Tissue/Condition
1	armstrong-v1	Affy	1081	72	Human	Blood
2	armstrong-v2	Affy	2194	72	Human	Blood
3	bhattacharjee	Affy	1543	203	Human	Lung
4	chowdary	Affy	182	104	Human	Breast, Colon
5	dyrskjot	Affy	1203	40	Human	Bladder
6	gordon	Affy	1626	181	Human	Lung
7	laiho	Affy	2202	37	Human	Colon
8	nutt-v1	Affy	1377	50	Human	Brain
9	nutt-v2	Affy	1070	28	Human	Brain
10	nutt-v3	Affy	1152	22	Human	Brain
11	pomeroy-v1	Affy	857	34	Human	Brain
12	pomeroy-v2	Affy	1379	42	Human	Brain
13	ramaswamy	Affy	1363	190	Human	Multi-tissue
14	shipp	Affy	798	77	Human	Blood
15	singh	Affy	339	102	Human	Prostate
16	su	Affy	1571	174	Human	Multi-tissue
17	west	Affy	1198	49	Human	Breast
18	yeoh-v1	Affy	2526	248	Human	Bone marrow
19	alpha factor	cDNA	1099	18	Yeast	Cell cycle synchronisation
20	cdc 15	cDNA	1086	24	Yeast	Cell cycle synchronisation
21	cdc 28	cDNA	1044	17	Yeast	Cell cycle synchronisation
22	elutriation	cDNA	935	14	Yeast	Cell cycle synchronisation
23	1mM menadione	cDNA	1050	9	Yeast	Environmental modifications
24	1M sorbitol	cDNA	1030	7	Yeast	Environmental modifications
25	15mM diamide	cDNA	1038	8	Yeast	Environmental modifications
26	25mM DTT	cDNA	991	8	Yeast	Environmental modifications
27	constant 32nM H2O2	cDNA	976	10	Yeast	Environmental modifications
28	diauxic shift	cDNA	1016	7	Yeast	Environmental modifications
29	complete DTT	cDNA	962	7	Yeast	Environmental modifications
30	heat shock 1	cDNA	988	8	Yeast	Environmental modifications
31	heat shock 2	cDNA	999	7	Yeast	Environmental modifications
32	nitrogen depletion	cDNA	1011	10	Yeast	Environmental modifications
33	YPD 1	cDNA	1011	12	Yeast	Environmental modifications
34	YPD 2	cDNA	1022	10	Yeast	Environmental modifications
35	Yeast sporulation	cDNA	1006	7	Yeast	Sporulation

### Experimental setup

Our objective is to assess to which extent biclustering algorithms and our own K-CPGC approach are able to find biclusters representative of the controlled conditions in our evaluation study. To do so, we analyze the gene subsets found by each approach and we check which of them are significantly enriched.

To compare all approaches on a fair basis, we look for (up to) *K*=10 biclusters for each controlled experiment. As detailed below, some algorithms do not produce so many solutions while others, including K-CPGC, could be tuned to produce more solutions. Ten biclusters from each data matrix are also considered as reasonable for the subsequent biological interpretation of the results.

All algorithms used in this work are available through R packages: *biclust* [30], *isa2* [31] and https://github.com/vbranders/mssm for K-CPGC. By default, the control parameters of each biclustering algorithm are those recommended by their original authors. For example, as proposed by the authors of CCA, the original data matrices are initially multiplied by 100 to match the range of data values their control parameters are assuming. The discretization step of xMOTIFs is performed with 10 equally spaced intervals from minimum to maximum. The K-CPGC threshold *θ* (see Interpretation within Problem definition) is set to the 75th percentile of expression values, specifically to each dataset. We consider such a threshold as representative of the objective of capturing high expression patterns. Given the performances of the CPGC approach on larger datasets, the K-CPGC method waits for convergence of the CPGC method. In other words, each call to the CPGC method is interrupted whenever the solution is proved optimal, or the best solution has not been improved for 10 s. We additionally compare the performances of the CPGC subroutine to the other approaches.

### Evaluation

In order to evaluate the biological relevance of the biclusters returned by the various algorithms in this study, a gene enrichment analysis is performed from the selected genes in each bicluster. Specifically, we perform an enrichment step for the selected genes through the Gene Ontology (GO; considering the Biological Process Ontology) [32] using the *clusterProfiler* R package [22].

For each of the 35 datasets, each of the 8 algorithms produces up to 10 biclusters. For each bicluster, the enrichment step provides a list of GO terms and FDR corrected *p*-values [33]. This *p*-value refers to the probability of selecting at random *n* genes out of the *N* genes from the original expression matrix, with *c* out of *n* being associated to the same functional class *C*. Let *s* be the true proportion of the *N* genes associated to the functional class *C*, the *p*-value associated to a GO term, or functional class *C*, is computed as:

3 $$\begin{array}{*{20}l} \text{Pr(} c \vert N, s, n \text{)} = \frac{ {{sN}\choose{c}} {{(1-s)N}\choose{n-c}} }{ {{N}\choose{n}}} \enspace. \end{array} $$

For each GO term, or functional class *C*, we calculate the *p*-value of the current submatrix enrichment as the probability of selecting at random at least *c* genes of this functional class *C* in the submatrix, where *c* is the actual number of genes from this class present in the current submatrix [19]. The smaller the *p*-values of the terms associated with a submatrix, the more likely the selected genes come from the same biological process.

According to the methodology proposed in [6, 7, 19, 20], a specific *bicluster* is considered *enriched* if there is at least one GO term with a FDR corrected *p*-value below 5%. An algorithm is considered better if it produces more enriched biclusters.

A refined analysis has also been proposed in [6, 34] through pairwise comparison of the smallest *p*-value among the GO terms found from the selected genes returned by each algorithm. Such a comparison could be criticized as it is limited to a single *p*-value for each algorithm, not necessarily computed for comparable GO terms. Instead, when comparing two algorithms *A*_1_ and *A*_2_, for any GO term considered significantly enriched (FDR corrected *p*-value < 5%) by both algorithms, one computes a performance difference as:

4 $$\begin{array}{*{20}l} \text{diff}(A_{1}, A_{2}) = -\log\left(\frac{p_{A_{1}}}{p_{A_{2}}}\right)  \end{array} $$

The larger diff(*A*_1_,*A*_2_), the smaller the corrected *p*-value of $p_{A_{1}}$ compared to $p_{A_{2}}$ with a positive difference whenever *A*_1_ outperforms *A*_2_.

## Supplementary information

**Additional file 1** Gene subsets identified by K-CPGC. Additional_file_1.pdf provides tables of the 20 most enriched GO terms identified by K-CPGC on *Saccharomyces cerevisiae* samples from 17 different conditions. One table is provided per condition.

**Additional file 2** Evolution of the number of gO terms identified. Additional_file_2.pdf illustrates the evolution of the number of enriched biclusters and enriched GO terms as *K* increases.

## Abbreviations

CCA: Cheng and Church’s algorithm
CP: Constraint programming
CPGC: Constraint programming with global constraint
FDR: False discovery rate
GO: Gene ontology
ISA: Iterative signature algorithm
MSSM: Max-sum submatrix
QUBIC: Qualitative biclustering

## Footnotes

1 All reports of time in the present work are computed from experiments on a MacBook Pro (OS version 10.10.5) laptop (Intel i7-2720 CPU 2.20-3.30GHz, 1GB RAM per run) with a single thread.
