# Supplementary material for: Identifying gene-specific subgroups: an alternative to biclustering
Source: BMC Bioinformatics. 2019 Dec 3;20:625. doi: 10.1186/s12859-019-3289-0 (PMC6888937; doi:10.1186/s12859-019-3289-0)
Supplement: Supplementary file 1 — Additional file 1 Gene subsets identified by K-CPGC. Additional_file_1.pdf provides tables of the 20 most enriched GO terms identified by K-CPGC on Saccharomyces cerevisiae samples from 17 different conditions. One table is provided per condition. [file 12859_2019_3289_MOESM1_ESM.pdf]

Additional File 1 - Analysis of the enriched GO terms identified by `K-CPGC`

Vincent Branders, Pierre Schaus, Pierre Dupont

December 26, 2018

This document presents the most significantly enriched Gene Ontology [1] (GO) terms identified by our approach on various datasets. Our approach, `K-CPGC`, and the 17 *Saccharomyces cerevisae* datasets [2] we study are described in the main manuscript. `K-CPGC` produces up to 10 submatrices of maximal sum per dataset. Each submatrix is associated with a subset of genes and a subset of samples. Up to 10 gene subsets are identified by `K-CPGC` per dataset. An enrichment step provides a list of GO terms and FDR (false discovery rate) corrected p-values [3] for each gene subset. The enrichment procedure is performed using the *clusterProfiler* R package [4].

The 20 most significantly enriched GO terms are reported in tables below. Each table presents the results of one dataset. One GO term is listed per row and the associated rank is provided in the first column. The second column gives the unique GO identifier of a term. The third column contains a short description for each term. Finally, the corrected p-value of the enrichment step is given in the last column. These tables confirm that identified GO terms are consistent with the controlled conditions under which these experiments were conducted.

The GO terms identified in the first four datasets, representative of cell cycles, are associated with some form of biogenesis, including ribosome, RNA, peptide and macromolecules synthesis. The GO terms identified in the next 12 datasets are indeed associated with various forms of response to stress-induced environments, including many representatives of the response to the stimulus, oxidation-reduction processes, and cellular responses to stress. Some GO terms also refer to generic responses to stress which are less specific to the controlled condition. For example, in the *complete DTT* dataset, many GO terms relate to alteration in the general patterns of protein biosyntheses as reported by Miller et al. [5]. The last experiment related to yeast sporulation includes GO terms referring to cell cycle, sporulation, and reproductive processes.

Author details

References

1. Ashburner, M., Ball, C.A., Blake, J.A., Botstein, D., Butler, H., Cherry, J.M., Davis, A.P., Dolinski, K., Dwight, S.S., Eppig, J.T., *et al.*: Gene ontology: tool for the unification of biology. Nature genetics **25**(1), 25 (2000)
2. Jaskowiak, P.A., Campello, R.J., Costa Filho, I.G.: Proximity measures for clustering gene expression microarray data: a validation methodology and a comparative analysis. IEEE/ACM Transactions on Computational Biology and Bioinformatics (TCBB) **10**(4), 845–857 (2013)
3. Benjamini, Y., Hochberg, Y.: Controlling the false discovery rate: a practical and powerful approach to multiple testing. Journal of the royal statistical society. Series B (Methodological), 289–300 (1995)
4. Yu, G., Wang, L.-G., Han, Y., He, Q.-Y.: clusterprofiler: an r package for comparing biological themes among gene clusters. OMICS: A Journal of Integrative Biology **16**(5), 284–287 (2012). doi:10.1089/omi.2011.0118
5. Miller, M.J., Xuong, N.-H., Geiduschek, E.P.: A response of protein synthesis to temperature shift in the yeast saccharomyces cerevisiae. Proceedings of the National Academy of Sciences **76**(10), 5222–5225 (1979)

Table 1: 01_alpha_factor.

	term	description	p-adjust.
1	GO:0042254	ribosome biogenesis	1.191613e-17
2	GO:0022613	ribonucleoprotein complex biogenesis	2.715624e-16
3	GO:0006364	rRNA processing	3.099617e-13
4	GO:0016072	rRNA metabolic process	3.099617e-13
5	GO:0034470	ncRNA processing	2.532992e-12
6	GO:0042274	ribosomal small subunit biogenesis	6.232817e-12
7	GO:0034660	ncRNA metabolic process	9.263555e-11
8	GO:0006396	RNA processing	4.683446e-10
9	GO:0000462	maturation of SSU-rRNA from tricistronic rRNA transcript	5.519431e-10
10	GO:0030490	maturation of SSU-rRNA	1.055367e-09
11	GO:0044085	cellular component biogenesis	6.271420e-08
12	GO:0000460	maturation of 5.8S rRNA	9.602590e-08
13	GO:0000466	maturation of 5.8S rRNA from tricistronic rRNA transcript	9.602590e-08
14	GO:0000478	endonucleolytic cleavage involved in rRNA processing	3.796748e-07
15	GO:0000479	endonucleolytic cleavage of tricistronic rRNA transcript	3.796748e-07
16	GO:0050801	ion homeostasis	4.712789e-07
17	GO:0055080	cation homeostasis	4.712789e-07
18	GO:0000469	cleavage involved in rRNA processing	8.120976e-07
19	GO:0044764	multi-organism cellular process	1.170023e-06
20	GO:0000746	conjugation	1.170023e-06

Table 2: 02_cdc_15.

	term	description	p-adjust.
1	GO:0042254	ribosome biogenesis	1.171585e-25
2	GO:0022613	ribonucleoprotein complex biogenesis	8.445391e-23
3	GO:0034660	ncRNA metabolic process	8.445391e-23
4	GO:0034470	ncRNA processing	1.209137e-22
5	GO:0006364	rRNA processing	2.809275e-22
6	GO:0016072	rRNA metabolic process	2.809275e-22
7	GO:0006396	RNA processing	4.656052e-19
8	GO:0016070	RNA metabolic process	4.518930e-17
9	GO:0090304	nucleic acid metabolic process	1.573745e-15
10	GO:0044085	cellular component biogenesis	8.007472e-15
11	GO:0010467	gene expression	6.087563e-12
12	GO:0042274	ribosomal small subunit biogenesis	1.510783e-11
13	GO:0006139	nucleobase-containing compound metabolic process	1.371075e-09
14	GO:0042273	ribosomal large subunit biogenesis	2.956637e-09
15	GO:0046483	heterocycle metabolic process	9.716562e-09
16	GO:0006725	cellular aromatic compound metabolic process	1.529971e-08
17	GO:0000460	maturation of 5.8S rRNA	1.955398e-08
18	GO:0000466	maturation of 5.8S rRNA from tricistronic rRNA transcript	1.955398e-08
19	GO:0030490	maturation of SSU-rRNA	1.955398e-08
20	GO:0000462	maturation of SSU-rRNA from tricistronic rRNA transcript	3.623493e-08

Table 3: 03_cdc_28.

	term	description	p-adjust.
1	GO:0002181	cytoplasmic translation	2.553447e-29
2	GO:0006412	translation	3.901098e-27
3	GO:0043043	peptide biosynthetic process	3.901098e-27
4	GO:0043604	amide biosynthetic process	3.901098e-27
5	GO:0006518	peptide metabolic process	8.745269e-25
6	GO:0043603	cellular amide metabolic process	2.993049e-23
7	GO:1901566	organonitrogen compound biosynthetic process	4.118333e-20
8	GO:0042254	ribosome biogenesis	1.682898e-16
9	GO:1901564	organonitrogen compound metabolic process	1.951916e-16
10	GO:0019538	protein metabolic process	1.482145e-13
11	GO:0022613	ribonucleoprotein complex biogenesis	2.570928e-12
12	GO:0044267	cellular protein metabolic process	2.730851e-12
13	GO:0042274	ribosomal small subunit biogenesis	5.359186e-10
14	GO:0006364	rRNA processing	2.515863e-09
15	GO:0042273	ribosomal large subunit biogenesis	5.502242e-08
16	GO:0016072	rRNA metabolic process	5.567943e-08
17	GO:0044271	cellular nitrogen compound biosynthetic process	3.490743e-07
18	GO:0034645	cellular macromolecule biosynthetic process	4.093896e-07
19	GO:0009059	macromolecule biosynthetic process	5.144026e-07
20	GO:0000462	maturation of SSU-rRNA from tricistronic rRNA transcript	9.260947e-07

Table 4: 04_elutriation.

	term	description	p-adjust.
1	GO:0042254	ribosome biogenesis	5.720403e-34
2	GO:0022613	ribonucleoprotein complex biogenesis	2.835895e-31
3	GO:0034660	ncRNA metabolic process	6.707776e-28
4	GO:0034470	ncRNA processing	1.025721e-25
5	GO:0006364	rRNA processing	1.627219e-24
6	GO:0016072	rRNA metabolic process	2.300636e-24
7	GO:0010467	gene expression	4.712617e-23
8	GO:0006807	nitrogen compound metabolic process	1.210243e-21
9	GO:0034641	cellular nitrogen compound metabolic process	3.630761e-20
10	GO:0006396	RNA processing	7.580284e-20
11	GO:0042274	ribosomal small subunit biogenesis	5.847937e-17
12	GO:0044085	cellular component biogenesis	9.716219e-17
13	GO:0016070	RNA metabolic process	1.620486e-16
14	GO:0006139	nucleobase-containing compound metabolic process	3.841940e-16
15	GO:0046483	heterocycle metabolic process	1.615821e-15
16	GO:0006725	cellular aromatic compound metabolic process	2.396934e-15
17	GO:0090304	nucleic acid metabolic process	2.418670e-15
18	GO:1901360	organic cyclic compound metabolic process	2.720483e-15
19	GO:0030490	maturation of SSU-rRNA	4.493421e-15
20	GO:0000462	maturation of SSU-rRNA from tricistronic rRNA transcript	1.435419e-14

Table 5: 05_1mM_menadione.

	term	description	p-adjust.
1	GO:0032196	transposition	4.575689e-13
2	GO:0032197	transposition, RNA-mediated	4.575689e-13
3	GO:0006979	response to oxidative stress	5.485371e-09
4	GO:0034599	cellular response to oxidative stress	2.435800e-08
5	GO:0055114	oxidation-reduction process	4.793730e-08
6	GO:0044710	single-organism metabolic process	3.606023e-07
7	GO:0042221	response to chemical	1.670056e-04
8	GO:0002181	cytoplasmic translation	0.001291574
9	GO:0019725	cellular homeostasis	1.889669e-03
10	GO:0070887	cellular response to chemical stimulus	2.766072e-03
11	GO:0006623	protein targeting to vacuole	2.766072e-03
12	GO:0072665	protein localization to vacuole	2.766072e-03
13	GO:0072666	establishment of protein localization to vacuole	2.766072e-03
14	GO:0006575	cellular modified amino acid metabolic process	3.421278e-03
15	GO:0008652	cellular amino acid biosynthetic process	3.421278e-03
16	GO:0045454	cell redox homeostasis	1.143711e-02
17	GO:0046394	carboxylic acid biosynthetic process	1.296318e-02
18	GO:0006520	cellular amino acid metabolic process	1.296318e-02
19	GO:0016053	organic acid biosynthetic process	1.296318e-02
20	GO:0006820	anion transport	1.296318e-02

Table 6: 06_1M_sorbitol.

	term	description	p-adjust.
1	GO:0009056	catabolic process	7.574141e-08
2	GO:0006508	proteolysis	7.574141e-08
3	GO:0044710	single-organism metabolic process	7.676728e-08
4	GO:1901575	organic substance catabolic process	9.198719e-08
5	GO:0044723	single-organism carbohydrate metabolic process	8.168864e-07
6	GO:0044763	single-organism cellular process	6.135262e-06
7	GO:0006091	generation of precursor metabolites and energy	6.135262e-06
8	GO:0030163	protein catabolic process	6.135262e-06
9	GO:0050896	response to stimulus	6.135262e-06
10	GO:0044257	cellular protein catabolic process	7.282433e-06
11	GO:0055114	oxidation-reduction process	9.709549e-06
12	GO:0044248	cellular catabolic process	1.761001e-05
13	GO:0006796	phosphate-containing compound metabolic process	2.396838e-05
14	GO:0006793	phosphorus metabolic process	2.636866e-05
15	GO:0005975	carbohydrate metabolic process	3.061083e-05
16	GO:0051603	proteolysis involved in cellular protein catabolic process	3.065663e-05
17	GO:0006950	response to stress	3.753313e-05
18	GO:0051716	cellular response to stimulus	3.755634e-05
19	GO:0044262	cellular carbohydrate metabolic process	4.816775e-05
20	GO:0015980	energy derivation by oxidation of organic compounds	6.838967e-05

Table 7: 07_15mM_diamide.

	term	description	p-adjust.
1	GO:0006457	protein folding	1.846434e-07
2	GO:0006081	cellular aldehyde metabolic process	2.444289e-07
3	GO:0055114	oxidation-reduction process	1.143481e-06
4	GO:0033554	cellular response to stress	1.746171e-06
5	GO:0044710	single-organism metabolic process	1.746171e-06
6	GO:0044712	single-organism catabolic process	1.746171e-06
7	GO:0070887	cellular response to chemical stimulus	2.990616e-06
8	GO:0006950	response to stress	2.990616e-06
9	GO:0050896	response to stimulus	5.417786e-06
10	GO:0042221	response to chemical	7.577489e-06
11	GO:0006979	response to oxidative stress	7.577489e-06
12	GO:0010035	response to inorganic substance	7.577489e-06
13	GO:0051716	cellular response to stimulus	7.938822e-06
14	GO:1901575	organic substance catabolic process	1.074632e-05
15	GO:0009056	catabolic process	1.074632e-05
16	GO:0005975	carbohydrate metabolic process	1.144059e-05
17	GO:0044723	single-organism carbohydrate metabolic process	4.072123e-05
18	GO:0034599	cellular response to oxidative stress	4.072123e-05
19	GO:0044282	small molecule catabolic process	9.929611e-05
20	GO:1901700	response to oxygen-containing compound	1.384823e-04

Table 8: 08_25mM_DTT.

	term	description	p-adjust.
1	GO:0002181	cytoplasmic translation	2.571265e-26
2	GO:0006412	translation	1.262146e-20
3	GO:0043043	peptide biosynthetic process	1.814313e-20
4	GO:0043604	amide biosynthetic process	1.270097e-19
5	GO:0006518	peptide metabolic process	4.110698e-19
6	GO:0043603	cellular amide metabolic process	3.413801e-18
7	GO:1901566	organonitrogen compound biosynthetic process	7.571918e-16
8	GO:0010467	gene expression	1.406438e-15
9	GO:0044260	cellular macromolecule metabolic process	5.610111e-14
10	GO:0022613	ribonucleoprotein complex biogenesis	2.348392e-13
11	GO:0044267	cellular protein metabolic process	2.376363e-13
12	GO:0034641	cellular nitrogen compound metabolic process	1.072584e-12
13	GO:0042254	ribosome biogenesis	1.369765e-12
14	GO:0043170	macromolecule metabolic process	1.769892e-12
15	GO:0019538	protein metabolic process	2.764025e-12
16	GO:0006807	nitrogen compound metabolic process	6.721745e-12
17	GO:1901564	organonitrogen compound metabolic process	1.907148e-11
18	GO:0009059	macromolecule biosynthetic process	1.565513e-10
19	GO:0006364	rRNA processing	1.763186e-10
20	GO:0016072	rRNA metabolic process	1.763186e-10

Table 9: 09_constant_32nM_H2O2.

	term	description	p-adjust.
1	GO:0055114	oxidation-reduction process	6.602138e-14
2	GO:0044710	single-organism metabolic process	2.692915e-09
3	GO:0006979	response to oxidative stress	8.271084e-08
4	GO:0034599	cellular response to oxidative stress	1.049329e-06
5	GO:0042221	response to chemical	1.246732e-05
6	GO:0019725	cellular homeostasis	1.246732e-05
7	GO:0044699	single-organism process	1.246732e-05
8	GO:0048878	chemical homeostasis	1.918285e-05
9	GO:0055072	iron ion homeostasis	2.170300e-05
10	GO:0070887	cellular response to chemical stimulus	2.460483e-05
11	GO:0042592	homeostatic process	2.804128e-05
12	GO:0098771	inorganic ion homeostasis	5.681460e-05
13	GO:0050801	ion homeostasis	7.432589e-05
14	GO:0055080	cation homeostasis	1.652456e-04
15	GO:0044723	single-organism carbohydrate metabolic process	1.716979e-04
16	GO:0044262	cellular carbohydrate metabolic process	2.412582e-04
17	GO:0055065	metal ion homeostasis	2.412582e-04
18	GO:0005975	carbohydrate metabolic process	2.862163e-04
19	GO:0010035	response to inorganic substance	2.862163e-04
20	GO:0055076	transition metal ion homeostasis	3.468919e-04

Table 10: 10_diauxic_shift.

	term	description	p-adjust.
1	GO:0015980	energy derivation by oxidation of organic compounds	7.076662e-17
2	GO:0006091	generation of precursor metabolites and energy	7.076662e-17
3	GO:0055114	oxidation-reduction process	3.610217e-16
4	GO:0045333	cellular respiration	1.631480e-13
5	GO:0044710	single-organism metabolic process	4.596238e-12
6	GO:0009060	aerobic respiration	1.173179e-11
7	GO:0044723	single-organism carbohydrate metabolic process	8.367914e-11
8	GO:0044262	cellular carbohydrate metabolic process	5.735060e-10
9	GO:0005975	carbohydrate metabolic process	2.338722e-09
10	GO:0044763	single-organism cellular process	1.133426e-08
11	GO:1901566	organonitrogen compound biosynthetic process	1.229324e-07
12	GO:0006099	tricarboxylic acid cycle	2.278109e-07
13	GO:0006412	translation	2.830024e-07
14	GO:0043043	peptide biosynthetic process	2.830024e-07
15	GO:0006518	peptide metabolic process	3.950002e-07
16	GO:0043604	amide biosynthetic process	3.950002e-07
17	GO:0006119	oxidative phosphorylation	5.075348e-07
18	GO:0043603	cellular amide metabolic process	1.018864e-06
19	GO:1901564	organonitrogen compound metabolic process	1.018864e-06
20	GO:0022900	electron transport chain	1.136910e-06

Table 11: 11_complete_DTT.

	term	description	p-adjust.
1	GO:0002181	cytoplasmic translation	6.924363e-59
2	GO:0006412	translation	2.772677e-55
3	GO:0043043	peptide biosynthetic process	5.084914e-54
4	GO:0010467	gene expression	1.800433e-52
5	GO:0043604	amide biosynthetic process	1.567400e-51
6	GO:0006518	peptide metabolic process	1.045339e-49
7	GO:0043603	cellular amide metabolic process	3.758523e-45
8	GO:1901566	organonitrogen compound biosynthetic process	2.466839e-40
9	GO:0022613	ribonucleoprotein complex biogenesis	9.607187e-40
10	GO:0042254	ribosome biogenesis	1.661256e-39
11	GO:0044271	cellular nitrogen compound biosynthetic process	6.496315e-38
12	GO:0034645	cellular macromolecule biosynthetic process	8.025738e-37
13	GO:0009059	macromolecule biosynthetic process	3.615328e-36
14	GO:0034641	cellular nitrogen compound metabolic process	6.877468e-34
15	GO:0006807	nitrogen compound metabolic process	1.482335e-31
16	GO:0044260	cellular macromolecule metabolic process	2.894952e-31
17	GO:0043170	macromolecule metabolic process	2.081095e-30
18	GO:0044267	cellular protein metabolic process	5.374936e-30
19	GO:0044085	cellular component biogenesis	2.198896e-28
20	GO:0044249	cellular biosynthetic process	2.496072e-28

Table 12: 12_heat_shock_1.

	term	description	p-adjust.
1	GO:0044699	single-organism process	1.561494e-10
2	GO:0005975	carbohydrate metabolic process	1.905360e-10
3	GO:0044723	single-organism carbohydrate metabolic process	3.756275e-10
4	GO:0044262	cellular carbohydrate metabolic process	9.092422e-10
5	GO:0006091	generation of precursor metabolites and energy	1.459404e-09
6	GO:0044710	single-organism metabolic process	6.348805e-09
7	GO:0044712	single-organism catabolic process	1.075406e-07
8	GO:0015980	energy derivation by oxidation of organic compounds	1.075406e-07
9	GO:0055114	oxidation-reduction process	3.361147e-07
10	GO:0044763	single-organism cellular process	3.455244e-07
11	GO:0006950	response to stress	5.776457e-07
12	GO:0006508	proteolysis	5.776457e-07
13	GO:0009056	catabolic process	6.113607e-07
14	GO:0016052	carbohydrate catabolic process	6.113607e-07
15	GO:0044724	single-organism carbohydrate catabolic process	6.113607e-07
16	GO:0050896	response to stimulus	1.274111e-06
17	GO:1901575	organic substance catabolic process	2.070463e-06
18	GO:0051716	cellular response to stimulus	5.109371e-06
19	GO:0005996	monosaccharide metabolic process	5.109371e-06
20	GO:0072524	pyridine-containing compound metabolic process	5.109371e-06

Table 13: 13_heat_shock_2.

	term	description	p-adjust.
1	GO:0022613	ribonucleoprotein complex biogenesis	6.398150e-58
2	GO:0042254	ribosome biogenesis	1.965485e-54
3	GO:0010467	gene expression	1.192324e-52
4	GO:0034660	ncRNA metabolic process	1.155424e-41
5	GO:0034470	ncRNA processing	3.906884e-40
6	GO:0006396	RNA processing	1.528704e-39
7	GO:0006364	rRNA processing	1.848234e-38
8	GO:0044085	cellular component biogenesis	3.906063e-38
9	GO:0016072	rRNA metabolic process	9.596845e-38
10	GO:0034641	cellular nitrogen compound metabolic process	7.892055e-37
11	GO:0006807	nitrogen compound metabolic process	5.818113e-35
12	GO:0044260	cellular macromolecule metabolic process	2.021910e-28
13	GO:0006412	translation	3.239351e-28
14	GO:0043043	peptide biosynthetic process	1.131198e-27
15	GO:0043170	macromolecule metabolic process	1.257546e-27
16	GO:0016070	RNA metabolic process	1.834100e-26
17	GO:0002181	cytoplasmic translation	7.073856e-26
18	GO:0006518	peptide metabolic process	1.291624e-25
19	GO:0043604	amide biosynthetic process	3.967591e-25
20	GO:0090304	nucleic acid metabolic process	3.270826e-22

Table 14: 14_nitrogen_depletion.

	term	description	p-adjust.
1	GO:1901605	alpha-amino acid metabolic process	1.029080e-19
2	GO:0016053	organic acid biosynthetic process	3.325122e-19
3	GO:0046394	carboxylic acid biosynthetic process	3.325122e-19
4	GO:0008652	cellular amino acid biosynthetic process	8.232890e-19
5	GO:1901607	alpha-amino acid biosynthetic process	1.747650e-18
6	GO:0019752	carboxylic acid metabolic process	5.286085e-18
7	GO:0043436	oxoacid metabolic process	7.086621e-18
8	GO:0006082	organic acid metabolic process	7.887305e-18
9	GO:0044283	small molecule biosynthetic process	7.887305e-18
10	GO:0006520	cellular amino acid metabolic process	8.789373e-18
11	GO:0044281	small molecule metabolic process	7.013921e-17
12	GO:0044711	single-organism biosynthetic process	6.173953e-13
13	GO:0032197	transposition, RNA-mediated	1.140563e-12
14	GO:0032196	transposition	1.140563e-12
15	GO:0044763	single-organism cellular process	2.378619e-12
16	GO:0009066	aspartate family amino acid metabolic process	6.690499e-12
17	GO:0044710	single-organism metabolic process	8.359101e-12
18	GO:0009067	aspartate family amino acid biosynthetic process	4.292215e-10
19	GO:0009084	glutamine family amino acid biosynthetic process	1.397522e-07
20	GO:0009064	glutamine family amino acid metabolic process	2.663769e-07

Table 15: 15_YPD_1.

	term	description	p-adjust.
1	GO:0055114	oxidation-reduction process	4.824315e-08
2	GO:0015980	energy derivation by oxidation of organic compounds	6.532605e-08
3	GO:0045333	cellular respiration	1.978998e-07
4	GO:0006091	generation of precursor metabolites and energy	7.468324e-07
5	GO:0044710	single-organism metabolic process	1.152118e-06
6	GO:0044763	single-organism cellular process	1.929617e-06
7	GO:0009060	aerobic respiration	2.720481e-06
8	GO:0006119	oxidative phosphorylation	2.220305e-05
9	GO:0022904	respiratory electron transport chain	4.184641e-05
10	GO:0042773	ATP synthesis coupled electron transport	4.184641e-05
11	GO:0042775	mitochondrial ATP synthesis coupled electron transport	4.184641e-05
12	GO:0022900	electron transport chain	8.983699e-05
13	GO:0044723	single-organism carbohydrate metabolic process	1.763532e-04
14	GO:0019236	response to pheromone	0.0004331063
15	GO:0019953	sexual reproduction	0.0004331063
16	GO:0044703	multi-organism reproductive process	0.0004331063
17	GO:0051704	multi-organism process	0.0004737045
18	GO:0033554	cellular response to stress	4.773348e-04
19	GO:0000746	conjugation	0.0005125271
20	GO:0000747	conjugation with cellular fusion	0.0005125271

Table 16: 16_YPD_2.

	term	description	p-adjust.
1	GO:0015980	energy derivation by oxidation of organic compounds	2.818721e-12
2	GO:0006091	generation of precursor metabolites and energy	1.958345e-11
3	GO:0055114	oxidation-reduction process	3.189884e-11
4	GO:0045333	cellular respiration	9.617200e-11
5	GO:0009060	aerobic respiration	2.215004e-09
6	GO:0044723	single-organism carbohydrate metabolic process	2.096364e-07
7	GO:0044262	cellular carbohydrate metabolic process	3.787796e-07
8	GO:0044710	single-organism metabolic process	1.647733e-06
9	GO:0006119	oxidative phosphorylation	5.683543e-06
10	GO:0022900	electron transport chain	1.406935e-05
11	GO:0005975	carbohydrate metabolic process	1.446949e-05
12	GO:0006099	tricarboxylic acid cycle	3.202010e-05
13	GO:0022904	respiratory electron transport chain	6.942742e-05
14	GO:0042773	ATP synthesis coupled electron transport	6.942742e-05
15	GO:0042775	mitochondrial ATP synthesis coupled electron transport	6.942742e-05
16	GO:0016310	phosphorylation	9.188584e-05
17	GO:0072329	monocarboxylic acid catabolic process	6.187705e-04
18	GO:0032787	monocarboxylic acid metabolic process	8.357263e-04
19	GO:0070887	cellular response to chemical stimulus	1.097774e-03
20	GO:0007005	mitochondrion organization	1.384831e-03

Table 17: 17_yeast_sporulation.

	term	description	p-adjust.
1	GO:0051321	meiotic cell cycle	2.791587e-30
2	GO:0022402	cell cycle process	8.461740e-30
3	GO:0007049	cell cycle	1.126408e-29
4	GO:1903046	meiotic cell cycle process	1.089221e-28
5	GO:0044702	single organism reproductive process	1.089221e-28
6	GO:0000003	reproduction	8.439478e-26
7	GO:0022414	reproductive process	1.521625e-25
8	GO:0048646	anatomical structure formation involved in morphogenesis	5.579015e-23
9	GO:0030435	sporulation resulting in formation of a cellular spore	1.380838e-22
10	GO:0043934	sporulation	2.989946e-22
11	GO:0030154	cell differentiation	2.040089e-20
12	GO:0009653	anatomical structure morphogenesis	4.514985e-20
13	GO:0048856	anatomical structure development	4.514985e-20
14	GO:0000280	nuclear division	2.341920e-19
15	GO:0048285	organelle fission	2.341920e-19
16	GO:0032502	developmental process	8.174620e-17
17	GO:0044767	single-organism developmental process	8.174620e-17
18	GO:0048869	cellular developmental process	1.516129e-16
19	GO:0034293	sexual sporulation	1.053153e-14
20	GO:0043935	sexual sporulation resulting in formation of a cellular spore	1.053153e-14
