# Supplementary material for: Identifying gene-specific subgroups: an alternative to biclustering
Source: BMC Bioinformatics. 2019 Dec 3;20:625. doi: 10.1186/s12859-019-3289-0 (PMC6888937; doi:10.1186/s12859-019-3289-0)

Additional File 2 - Analysis of the enrichment as a function of K

Vincent Branders, Pierre Schaus, Pierre Dupont

December 26, 2018

This document presents the evolution of the Gene Ontology [1] (GO) enrichment as a function of K, the maximal number of biclusters to find. Our approach, `K-CPGC`, the six biclustering algorithms (`CCA`, `ISA`, `QUBIC`, `Plaid`, `Spectral` and `xMOTIFs`) and the 17 *Saccharomyces cerevisae* datasets [2] we consider are detailed in the main manuscript. A subset of genes and a subset of samples are defined for each bicluster identified. An enrichment step provides a list of GO terms and FDR (false discovery rate) corrected p-values [3] for each subset of genes. The enrichment procedure is performed using the *clusterProfiler* R package [4].

We compare algorithms on the number of enriched biclusters. According to the methodology proposed in [5, 6, 7, 8], a specific bicluster is considered enriched if there is at least one GO term with an FDR corrected p-value below 5%. As a supplementary evaluation, we compare algorithms on the number of GO terms enriched (with an FDR corrected p-value below 5%).

Fixing parameter K is required for appropriate evaluation of algorithms. Choosing a value, however, depends on the considered algorithm and dataset. In practice, this is not a critical choice since the analyst can start with $K = 1$ and use the proposed gene enrichment analysis to check whether the successive biclusters and GO terms returned by increasing K are still significantly enriched. Consequently, we report here the evolution of the number of enriched biclusters and the evolution of the number of enriched GO terms as a function of K. Value of parameter K ranges from 1 up to no significant improvement.

Each dataset is separately normalized by subtracting a threshold θ to all matrix entries before using `K-CPGC`. The threshold θ is set to the 75th percentile of expression values, specifically to each dataset, in the main manuscript. We consider such a threshold as representative of the objective of capturing high expression patterns. We also examine the 65th and 85th percentiles of expression values to complement analyzes of this document. We report as `K-CPGC_0_xx` the results of our approach after normalization through subtraction of the `xx`th percentile. Results reported as `K-CPGC` in the main manuscript correspond to results reported as `K-CPGC_0_75` in this document.

Figure 1 presents the evolution of the number of enriched biclusters as a function of K. We observe a rapidly growing number of enriched biclusters up to the chosen value in the main manuscript ($K = 10$). Our approach, with its three different θ values, essentially identifies more enriched biclusters than other approaches in the first part of the graph. Nevertheless, `CCA` produces more enriched biclusters in the long run. We explain the absence of important improvements in our approach by

the size of the discovered gene subsets. Indeed, biclusters are identified only when there is some signal remaining in the matrix. Therefore, identifying larger subsets of genes is penalized, as the remaining signal depends on the previously identified (and masked) biclusters. Table 1 presents the average size of gene subsets for $K = 10$ and $K = 40$. We observe that the difference between CCA and the three variants of K-CPGC increases with K. A data analyst would consider smaller values of K given the size of the datasets, the size of the identified subsets of genes and the results from Figure 1.

Table 1: Size of gene subsets averaged on 17 *Saccharomyces cerevisae* datasets.

Name	$K = 10$	$K = 40$
K-CPGC_0_65	129	98
K-CPGC_0_75	110	93
K-CPGC_0_85	81	69
CCA	84	40
ISA	82	82
QUBIC	170	122
Plaid	130	126
Spectral	6	6
xMOTIFs	38	17

Figure 2 presents the number of different enriched GO terms identified by each approach for increasing values of K. We observe a rapidly growing number of enriched GO terms up to the chosen value in the main manuscript ($K = 10$), for most approaches, as in Figure 1. It confirms the ability of K-CPGC to quickly identify a large part of the relevant signal. It is furthermore noticeable that K-CPGC_0_65 and K-CPGC_0_75 identify more GO terms than all others approaches for any value of the parameter K.

Interpretations of both graphs can be reunited by observing that:

1 K should be large enough to observe differences regarding the enrichment,

2 K should, however, be small enough to ensure that there still is a signal to be found, regarding the number of biclusters and the number of GO terms.

Author details

References

1. Ashburner, M., Ball, C.A., Blake, J.A., Botstein, D., Butler, H., Cherry, J.M., Davis, A.P., Dolinski, K., Dwight, S.S., Eppig, J.T., *et al.*: Gene ontology: tool for the unification of biology. Nature genetics **25**(1), 25 (2000)
2. Jaskowiak, P.A., Campello, R.J., Costa Filho, I.G.: Proximity measures for clustering gene expression microarray data: a validation methodology and a comparative analysis. IEEE/ACM Transactions on Computational Biology and Bioinformatics (TCBB) **10**(4), 845–857 (2013)
3. Benjamini, Y., Hochberg, Y.: Controlling the false discovery rate: a practical and powerful approach to multiple testing. Journal of the royal statistical society. Series B (Methodological), 289–300 (1995)
4. Yu, G., Wang, L.-G., Han, Y., He, Q.-Y.: clusterprofiler: an r package for comparing biological themes among gene clusters. OMICS: A Journal of Integrative Biology **16**(5), 284–287 (2012). doi:10.1089/omi.2011.0118
5. Padilha, V.A., Campello, R.J.: A systematic comparative evaluation of biclustering techniques. BMC bioinformatics **18**(1), 55 (2017)
6. Li, G., Ma, Q., Tang, H., Paterson, A.H., Xu, Y.: Qubic: a qualitative biclustering algorithm for analyses of gene expression data. Nucleic acids research **37**(15), 101–101 (2009)
7. Prelić, A., Bleuler, S., Zimmermann, P., Wille, A., Bühlmann, P., Gruissem, W., Hennig, L., Thiele, L., Zitzler, E.: A systematic comparison and evaluation of biclustering methods for gene expression data. Bioinformatics **22**(9), 1122–1129 (2006)
8. Eren, K., Deveci, M., Küçüktunç, O., Çatalyürek, Ü.V.: A comparative analysis of biclustering algorithms for gene expression data. Briefings in bioinformatics **14**(3), 279–292 (2012)

Figure 1: **Evolution of the number of enriched biclusters as a function of K.** The left axis presents the cumulated number of biclusters identified as K, the maximal number of biclusters to be found, increases. The right axis presents a performance computed as the number of enriched biclusters divided by the number of enriched biclusters identified by the best algorithm at $K = 40$. The 100% performance corresponds to identifying 142 enriched biclusters. Note that `K-CPGC_0_75` corresponds to `K-CPGC` in the main manuscript.

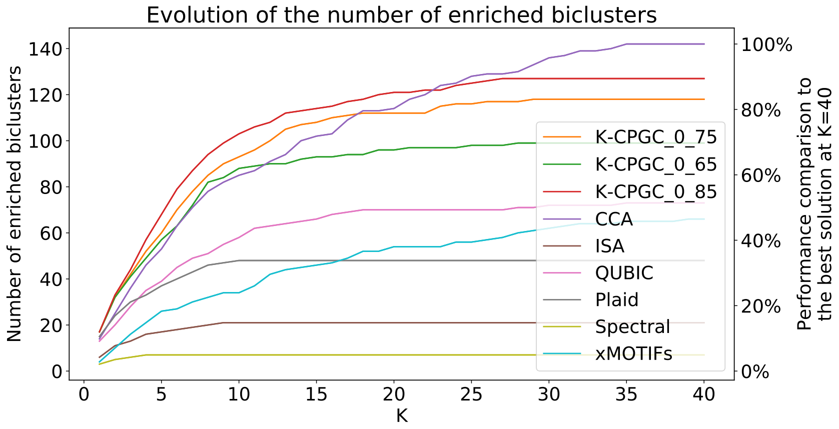

Figure 2: **Evolution of the number of enriched GO terms as a function of K.** The left axis presents the cumulated number of different GO terms identified as K, the maximal number of biclusters to be found, increases. The right axis presents a performance computed as the number of different enriched GO terms identified divided by the number of different enriched GO terms identified by the best algorithm at $K = 40$. The 100% performance corresponds to identifying 2879 enriched GO terms. Note that `K-CPGC_0_75` corresponds to `K-CPGC` in the main manuscript.

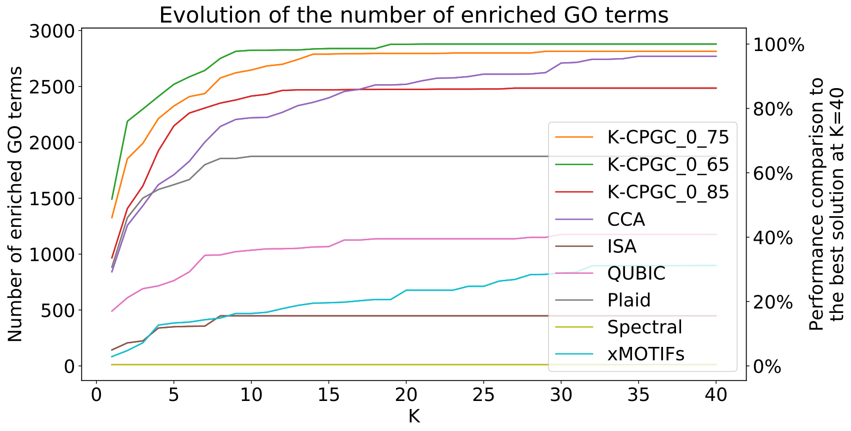

Supplement: Supplementary file 2 — Additional file 2 Evolution of the number of gO terms identified. Additional_file_2.pdf illustrates the evolution of the number of enriched biclusters and enriched GO terms as K increases. [file 12859_2019_3289_MOESM2_ESM.pdf]
